# Study on the relationships between the accumulation and translocation of dry matter and nitrogen and flower/pod development into seeds and seed yields in Chinese milk vetch

**DOI:** 10.1371/journal.pone.0278832

**Published:** 2023-03-23

**Authors:** Chunfeng Zheng, Chunzeng Liu, Chao Li, Chun Liu, Liangpeng Nie, Pengfei Shi, Jishi Zhang, Yuhu Lv, Jie Li, Weidong Cao

**Affiliations:** 1 Institute of Plant Nutrition Agricultural Resources and Environmental Sciences, Henan Academy of Agricultural Sciences, Zhengzhou, China; 2 Luoshan Institute of Agricultural Sciences, Luoshan, Xinyang, China; 3 Institute of Plant Nutrition Agricultural Resources and Environmental Sciences, Xinyang Academy of Agricultural Sciences, Xinyang, China; 4 Institute of Agriculture Resources and Regional Planning, Chinese Academy of Agricultural Sciences, Beijing, China; Feroze Gandhi Degree College, INDIA

## Abstract

Further improvements to the yield potential of Chinese milk vetch seed are essential for the planting demand of green manure. Flower and pod development directly determines the number of seeds and the seed yield of Chinese milk vetch. However, the accumulation and translocation of dry matter and nitrogen between plant organs directly affects flower and pod development and morphological formation. There are few studies that analyse the relationship between the accumulation and transport of dry matter and nitrogen and the number of flowers, pods, grains and seed yield during Chinese milk vetch’s critical development period. This study aimed to determine the seed yield response to dry matter and nitrogen accumulation and translocation during the Chinese milk vetch growth period and to quantify the relationship between these factors to predict Chinese milk vetch seed yield. Experiments were performed during the 2017–2018 and 2018–2019 growing seasons at the Dayuzhuang experimental field. The first experiment involved five foliar application stages (late wintering stage, returning green stage, squaring stage, pre-flowering stage, and 5 days after flowering) and six foliar application concentrations of borate solution (0, 500, 1000, 2000, 4000, and 6000 mg L^-1^). Experiment 2 included five foliar application stages (late wintering stage, returning green stage, squaring stage, pre-flowering stage, and 5 days after flowering) and six foliar application concentrations of paclobutrazol (0, 200, 300, 400, 500, and 600 mg L^-1^). When the dry matter mass in the full flowering stage was 3500–4500 kg hm^-2^, the seed yield reached more than 800 kg hm^-2^. When the translocated assimilates were stored in the vegetative organs before flowering, the assimilate translocation rate and their contributions to seed yield were 1500–1800 kg hm^-2^, 30–35%, and 28–38%, respectively, and the Chinese milk vetch seed yield was predicted to reach 800–1000 kg hm^-2^ at maturity. When the nitrogen translocation amount in the vegetative organs before flowering, the nitrogen translocation rate, and the contribution rate to the seed yield were 68–78 kg hm^-2^, 65–75%, and 75–85%, respectively, the Chinese milk vetch seed yield was predicted to reach 800–1000 kg hm^-2^ at maturity. If the accumulation and translocation index values of dry matter and nitrogen were lower or higher than the above ranges, the seed yield was lower than 800 kg hm^-2^. The results of this study revealed the mechanism by which dry matter and nitrogen accumulation and translocation affect the Chinese milk vetch seed yield. These findings enrich the seed yield formation theory of Chinese milk vetch. They provide an early determination and quantitative regulation of high and stable seed yield for Chinese milk vetch in the field and aid researchers to integrate multiple production technologies for the sustainable production of Chinese milk vetch.

## Introduction

Under the concept that clear waters and green mountains are invaluable assets in agricultural production, the adjustment of the planting industry structure, the improvement of farmland ecology, the combination of cultivated land use and maintenance, and the high-quality green development of agriculture will become the mainstays of China over the long term. Green manure plays a unique and effective role in the main strategic tasks of agriculture [1–3]. It plays a key role in improving medium- and low-yield fields and improving the quality of cultivated land, making it an important technical and material guarantee for good fields. It is an important means to support the strategy of reducing fertilizer application to major agricultural areas in China [2, 4, 5]. By using green manure to improve soil fertility and increase efficiency, reduce emissions, and restore the ecological environment, it is possible to allow for the rest and recuperation of cultivated land and the combination of farmland use and maintenance, thereby storing grain in land and technology [2, 6–10]. With the advancement of agricultural green development and the improved quality and protection of cultivated land in China, green manure planting and utilization have been increasingly adopted by producers as an important technical measure for their work.

With the area of green manure planting and use gradually increasing, the high-yield breeding of green manure seeds has become a key bottleneck in developing the green manure industry [11, 12]. Chinese milk vetch (*Astragalus sinicus* L.) (family Fabaceae) is one of the traditional green manure crops in China, with the role of improving the physical and chemical properties of soil, increasing the number and diversity of soil microorganisms, and improving soil fertility [13–15]. At present, the planting area of Chinese milk vetch accounts for 60% of the total green manure planting area in China, and the seed production of Chinese milk vetch has become an important link in developing green manure production [16, 17]. The Chinese milk vetch planting area in South Henan is the national Chinese milk vetch seed expansion base in China. However, due to the long flowering period of this plant, its inconsistent seed maturity period, its high rate of flower/pod shedding, its vigorous growth in the middle and late growth stages, the easy lodging of its stems, its lack of a nutrient supply at the late reproductive stage, the impact of its own seed-setting characteristics, and external climatic conditions, the seed yield is low, which seriously hurts the industrialization and promotion of Chinese milk vetch and becoming a limiting factor in the stable development of green manure production [16, 18–20].

The crop yield level is closely related to the accumulation and translocation of dry matter and nitrogen. Analysing the dynamics in the accumulation and translocation of dry matter and nitrogen during crop growth has important practical value in revealing the formation of crop yields [21–24]. The dry matter and nitrogen accumulation and the translocation processes of Chinese milk vetch are important factors that determine its seed yield [25]. However, to date, the relationship between dry matter and nitrogen accumulation and the translocation and seed yield and their effects on seed yield have not been reported. Pampana *et al*. [26] noted that improving grain legume yields requires either reduced N remobilization or an enhanced N supply; thus, a useful strategy is to add mineral N at flowering. Based on previous research, foliar spraying with different concentrations of borax fertilizer and plant growth retarder at different growth stages of Chinese milk vetch could result in different seed yields. Then, we further studied the seed yield relationship to the dry matter and nitrogen accumulation and the translocation of Chinese milk vetch during the critical period of flower/pod development. The purpose of this study was to clarify the seed yield response of Chinese milk vetch to its dry matter and nitrogen accumulation and its translocation during the growth period. The mechanism of dry matter and nitrogen accumulation and of translocation impacting the seed yield of Chinese milk vetch was revealed, and the relationship between them was quantified to devise a method for predicting the seed yield of Chinese milk vetch, to provide early diagnosis and quantitative regulation of high and stable yields of Chinese milk vetch and to integrate multiple production technologies to achieve the sustainable development of Chinese milk vetch production.

## Materials and methods

### General conditions

Experiments were performed outdoors during the 2017–2018 (experiment 1) and 2018–2019 (experiment 2) growing seasons in Dayuzhuang, Lanqing Township, Zhengyang County, Henan Province, China (32°16’N, 114°11’E). The test field soil type was lime concretion black soil, and the texture was clayey. The organic matter content within the top 20 cm of soil was 17.2 g kg^-1^, the total nitrogen was 0.9 g kg^-1^, the alkali-hydrolysis nitrogen was 102.98 mg kg^-1^, the available phosphorus was 28.7 mg kg^-1^, and the available potassium was 125.42 mg kg^-1^.

Immediately before sowing, all the experimental plots were fertilized with 187.5 kg ha^-1^ of compound fertilizer (N:P_2_O_5_:K_2_O = 24:11:10) as a basal application. Xinzi No. 1 was chosen as the experimental material. Seeds were sown by mixing them well with fine sand within the optimal sowing period for Chinese milk vetch in the region on 15 September 2017 and 17 September 2018. The plant density was 90 or 85 plants m^−2^ with an equal row spacing of 10 cm and uniform distribution in 2017–2018 or 2018–2019, respectively. Field management generally followed the local standard production practices for high-yielding Chinese milk vetch.

The area has a warm, temperate, semi-humid continental monsoon climate. The climatic conditions were highly similar between the 2017–2018 and 2018–2019 growing periods. During the entire growth season, the total accumulated temperatures were 1957°C and 1930°C in 2017–2018 and 2018–2019, respectively. The monthly average temperatures were 10.04°C and 10.02°C in 2017–2018 and 2018–2019, respectively. The annual total precipitation was 160.7 mm and 162.5 mm in 2017–2018 and 2018–2019, respectively. (Meteorological data of 2017–2019, provided by the Zhengyang Meteorological Bureau.).

### Treatments and experimental design

#### Experiment 1

Treatments consisted of a factorial combination of five foliar application stages (late wintering stage, returning green stage, squaring stage, pre-flowering stage, and 5 days after flowering) and six foliar application concentrations of borate solution (0, 500, 1000, 2000, 4000, and 6000 mg L^-1^). The timing was dependent on the critical plant growth and developmental stage as proposed by Zhong [25] Borate was applied as 99.5% borax (Na_2_B_4_O_7_·10H_2_O, containing 11.3% boron). Each plot spray volume was 750 kg hm^-2^. The protocol resulted in a layer of spray being deposited on the leaf surfaces without dripping. The amount and spraying standard of boron was therefore controlled using the method described by Zheng *et al*. [27].

The treatments were arranged into a completely randomized design with three replicates. In all, there were 90 plots, with each one being a microcrop used as an experimental unit, and each plot area was 20 m^2^.

#### Experiment 2

Treatments consisted of a factorial combination of five foliar application stages (late wintering stage, returning green stage, squaring stage, pre-flowering stage, and 5 days after flowering) and six foliar application concentrations of paclobutrazol (0, 200, 300, 400, 500, and 600 mg L^-1^). Paclobutrazol is a 15% wettable powder that is a commercial reagent provided by Anyang Quanfeng Biotechnology Co., Ltd. Each plot spray volume was 750 kg hm^-2^. The protocol resulted in a layer of spray being deposited on the leaf surfaces without dripping. The amount and spraying standard of paclobutrazol was therefore controlled by using the method described by Zheng *et al*. [16].

The treatments were arranged in a completely randomized design with three replicates. Again, there were 90 plots, each one being a microcrop used as an experimental unit, and each plot area was 20 m^2^.

### Sampling and measurements

#### Determination of nutrient content

During the key growth period of Chinese milk vetch, elongation, squaring, early flowering, full flowering, and maturity, 1 m^2^ of the aboveground plants were randomly selected from each plot. The stems, leaves, and inflorescences (including flower buds, flowers, and pods) were all weighed and fixed at 105°C for 30 min and then baked at 80°C until reaching a constant weight. The sampling period and sample pre-treatment were observed by using the method described by Gao *et al*. [28].The dry matter was weighed using an electronic balance with an accuracy of 0.0001 g, and each sample was crushed to retain for measurements. The nitrogen content was treated with H_2_SO_4_-H_2_O_2_ and was measured by Kjeldahl method according to Cox *et al*. [29]. The dry matter and nitrogen accumulation and translocation were calculated using the following formulas (Qian *et al*. [30]):

$$\text{VW}={\text{VW}}_{1}-{\text{VW}}_{2}$$

$$\text{TV}\%=\text{VW}/{\text{VW}}_{1}\times 100$$

CV%=VW/SW×100

where VW is the translocation amount of stored assimilate (dry matter) in the vegetative organs before flowering in kg hm^-2^; VW_1_ (kg hm^-2^) and VW_2_ (kg hm^-2^) are the dry weights in vegetative organs at the early flowering stage and the dry weight in vegetative organs at the maturity stage, respectively; TV% is the translocation rate of stored assimilates in the vegetative organs before flowering; and CV% is the contribution rate to the seed yield. VN = VN_1_-VN_2_

$$\text{TN}\%=\text{VN}/{\text{VN}}_{1}\times 100$$

CN%=VN/SN×100

where VN is the nitrogen translocation amount in vegetative organs before flowering in kg hm^-2^; VN_1_ (kg hm^-2^) and VN_2_ (kg hm^-2^) are the nitrogen accumulation amount in vegetative organs at the early flowering stage and the nitrogen accumulation amount in vegetative organs at the maturity stage, respectively; TN% is the translocation rate of the nitrogen accumulation amount in the vegetative organs before flowering; and CN% is the contribution rate to the seed yield.

#### Seed-setting characteristics investigation

**Fig 1:**
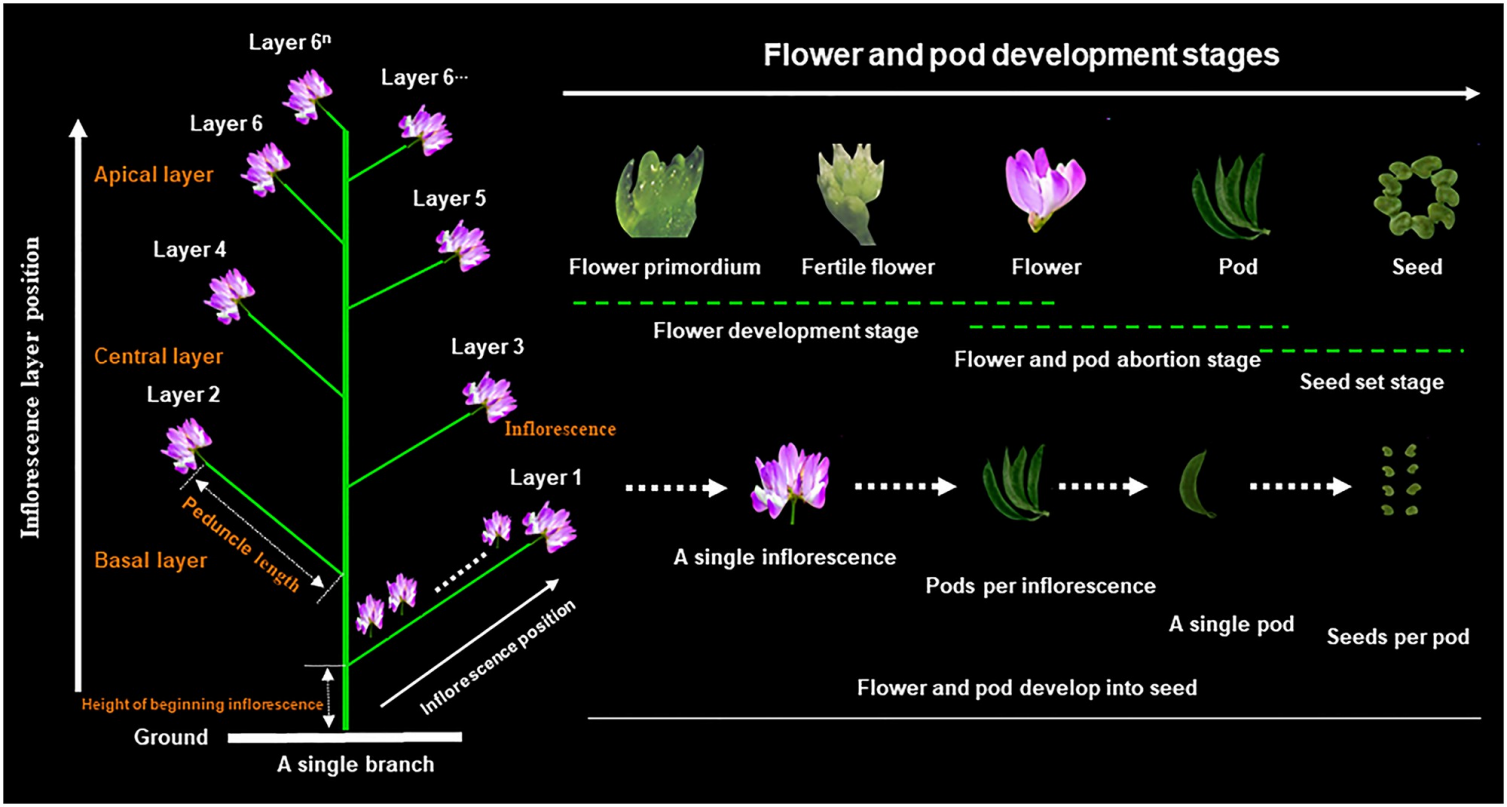
Morphological structure and the regularity of flower and pod development in Chinese milk vetch. These photographs were captured using a stereomicroscope (SZN-6 (45x), OPTIKA S.R.L., ITALY). The left panel illustrates the inflorescence layers (from bottom to top). The left bottom panel illustrates the inflorescence position of the inflorescence layers, and the inflorescences are numbered according to their positions relative to the rachis from most proximal to most distal. The panel on the upper right side illustrates the flower and pod developmental dynamics in three stages: the flower development stage (from the first appearance of the flower primordium to pollen maturity), flower and pod abortion stage (i.e., the fall-off stage of the flower and pod), and seed set stage (flower and pod develop into seed). The bottom right panel illustrates the processes of inflorescence development into seeds. The illustrations and photographs are not to scale; for reference, the flower widths are approximately 4.5 mm at pollen maturity, and the pod width and pod length are approximately 3 mm and 23 mm, respectively.

The morphological structure and flower/pod development process of Chinese milk vetch are shown in Fig 1. During the full flowering stage, 1 m^2^ of aboveground plants were randomly collected from each plot to count their flowers. During the maturity stage, 1 m^2^ of aboveground plants was randomly selected from each plot. The number of effective plants, first-grade branches, inflorescences, pods (seed pods, infertile pods (flat pods)), seeds, and seed weight were examined according to conventional methods, and 2 m^2^ of aboveground plants were harvested for seed yield calculations. The number of seeds per pod and the seed-setting rate of the pods were calculated according to Zheng *et al*. [27]:

Numberofseedsperpod=numberofseedsperunitarea/numberofpodsperunitarea

$$\text{Seed}\;\text{setting}\;\text{rate}\;\text{of}\;\text{the}\;\text{pod}\left(\%\right)=\text{number}\;\text{of}\;\text{seed}\;\text{pods}\;\text{per}\;\text{unit}\;\text{area}/\text{total}\;\text{number}\;\text{of}\;\text{pods}\;\text{per}\;\text{unit}\;\text{area}\times 100$$

### Statistical analysis

All the data were subjected to analysis of variance, considering the factorial combination of five foliar application stages and six foliar application concentrations of borate solution in Experiment 1 and the factorial combination of five foliar application stages and six foliar application concentrations of paclobutrazol in Experiment 2. The data were analysed in PASW (version 18.0) to examine the differences between the spraying treatments. Least significant differences were calculated at a probability level of *p* < 0.05. Figures were created using Microsoft Excel 2016.

## Results

### Correlation analysis of Chinese milk vetch seed yield and seed-setting factors

The correlation analysis on the seed yield of Chinese milk vetch and seed-setting factors showed that the number of inflorescences per unit area was positively correlated with the number of branches per unit area and the inflorescence number per first-grade branch (Table 1). The effective plant number per unit area was negatively correlated with the first-grade branch number per plant (Table 1). The seed number per pod was positively correlated with the seed setting pod rate (Table 1). The correlation coefficients of the seed yield with the number of inflorescences per unit area, first-grade branches and pods per effective inflorescence, the seed setting rate of pods, and the seed number per pod were all above 0.9, showing positive correlations (Table 1). There was little correlation between the seed yield and the number of plants, number of branches, and thousand-seed weight (Table 1). In summary, the seed yield of Chinese milk vetch is closely correlated with seed setting factors, such as the number of inflorescences and pods, the seed setting rate of pods, and the number of seeds, but not the number of plants, number of branches, or thousand-seed weight.

**Table 1:** Relevant analysis of seed yield and seed-setting factors under spraying treatments (experiments 1 and 2).

Variable	EPN (m^-2^)	1stBN (m^-2^)	IN (m^-2^)	1^st^ BNPP	INP 1^st^ B	PNPEI	SSPR	SNPP	1000-SW	Seed yield
EPN (m^-2^)	1									
1^st^ BN (m^-2^)	0.62	1								
IN (m^-2^)	0.65	0.75*	1							
1^st^ BNPP	-0.64*	0.77*	0.46	1						
INP 1^st^ B	0.025	0.037	0.79*	0.027	1					
PNPEI	0.032	0.043	0.45	0.028	0.54	1				
SSPR	0.021	0.030	0.58	0.035	0.55	0.69	1			
SNPP	0.057	0.053	0.65	0.032	0.63	0.69	0.70*	1		
1000-SW	0.47	0.40	0.50	0.41	0.48	0.47	0.46	0.67	1	
Seed yield	0.62	0.69	0.91**	0.67	0.90**	0.93**	0.95**	0.94**	0.62	1

*Note*: EPN—Effect plant number per m^2^; 1^st^ BN—First grade branch number per m^2^; IN—Inflorescence number per m^2^; 1^st^ BNPP—First grade branch number per plant; INP 1^st^ B—Inflorescence number per first grade branch; PNPEI—Pods number per effective inflorescence; SSPR—Seed-setting pod rate (%); SNPP—Seed number per pod; and SW—Seed weight.

*, ** represent the level of significance of the MS values (0.05 and 0.01, respectively).

### Relationships of dry matter accumulation to seed number and seed yield

**Fig 2:**
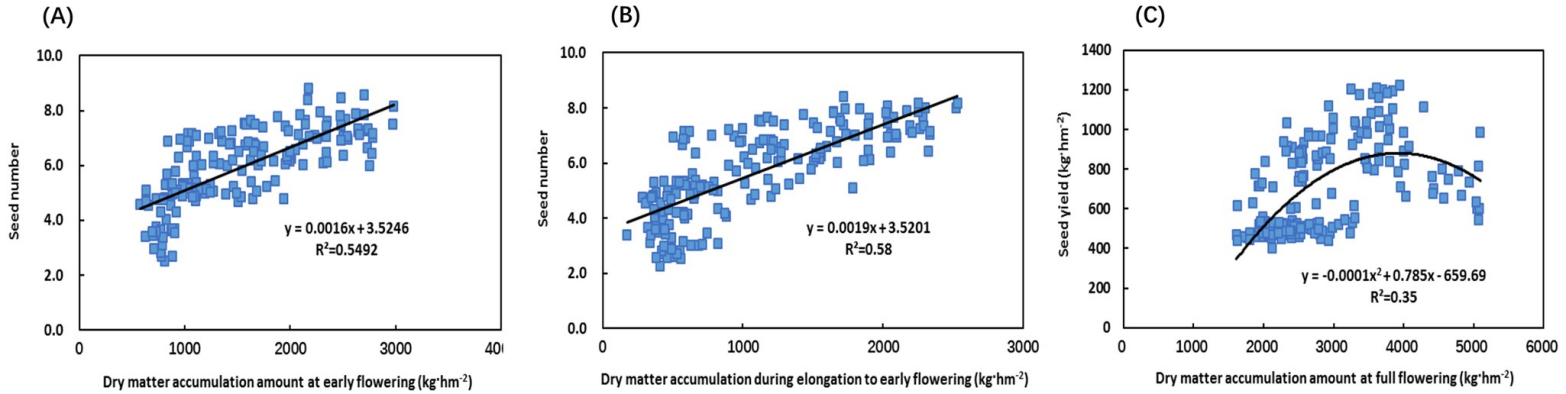
Relationships of dry matter accumulation with seed number and seed yield. Relationship between seed number and dry matter accumulation amount (dry matter accumulation amount at early flowering or elongation to early flowering, in experiments 1 and 2; n = 180, A and B, respectively) and the relationship between seed yield and dry matter accumulation amount at full flowering (in experiments 1 and 2; n = 180, C) of Chinese milk vetch.

The dry matter mass at the early flowering stage and the dry matter accumulation at the elongation stage to the early flowering stage showed a linear correlation with the seed number, and the seed number increased with the dry matter mass at the early flowering stage and at the elongation to the early flowering stage (Fig 2A and 2B). The coefficients of determination were 0.5492 and 0.58, respectively (*Y = 0*.*0016X + 3*.*5246*, *R*^*2*^ *= 0*.*5492*, *P<0*.*001*, *n = 180; Y = 0*.*0019X + 3*.*5201*, *R*^*2*^ *= 0*.*58*, *P<0*.*001*, *n = 180*) (Fig 2A and 2B). The relationship between seed yield at the maturity stage and dry matter mass at the full flowering stage was quadratic (Fig 2C). The seed yield first increased and then decreased with the increasing dry matter, with a coefficient of determination for the regression equation of 0.35 (*Y = -0*.*0001X*^*2*^ *+ 0*.*785X -659*.*69*, *R*^*2*^ *= 0*.*35*, *P<0*.*001*, *n = 180*) (Fig 2C). According to the prediction by the regression equation, when the dry matter totalled 3500–4500 kg hm^-2^ during the full flowering stage, a relatively high seed yield was obtained, and the seed yield reached more than 800 kg hm^-2^ (Fig 2C).

### Relationship between the dry matter and nitrogen accumulation in the inflorescence organs of Chinese milk vetch and the number of flowers and pods

**Fig 3:**
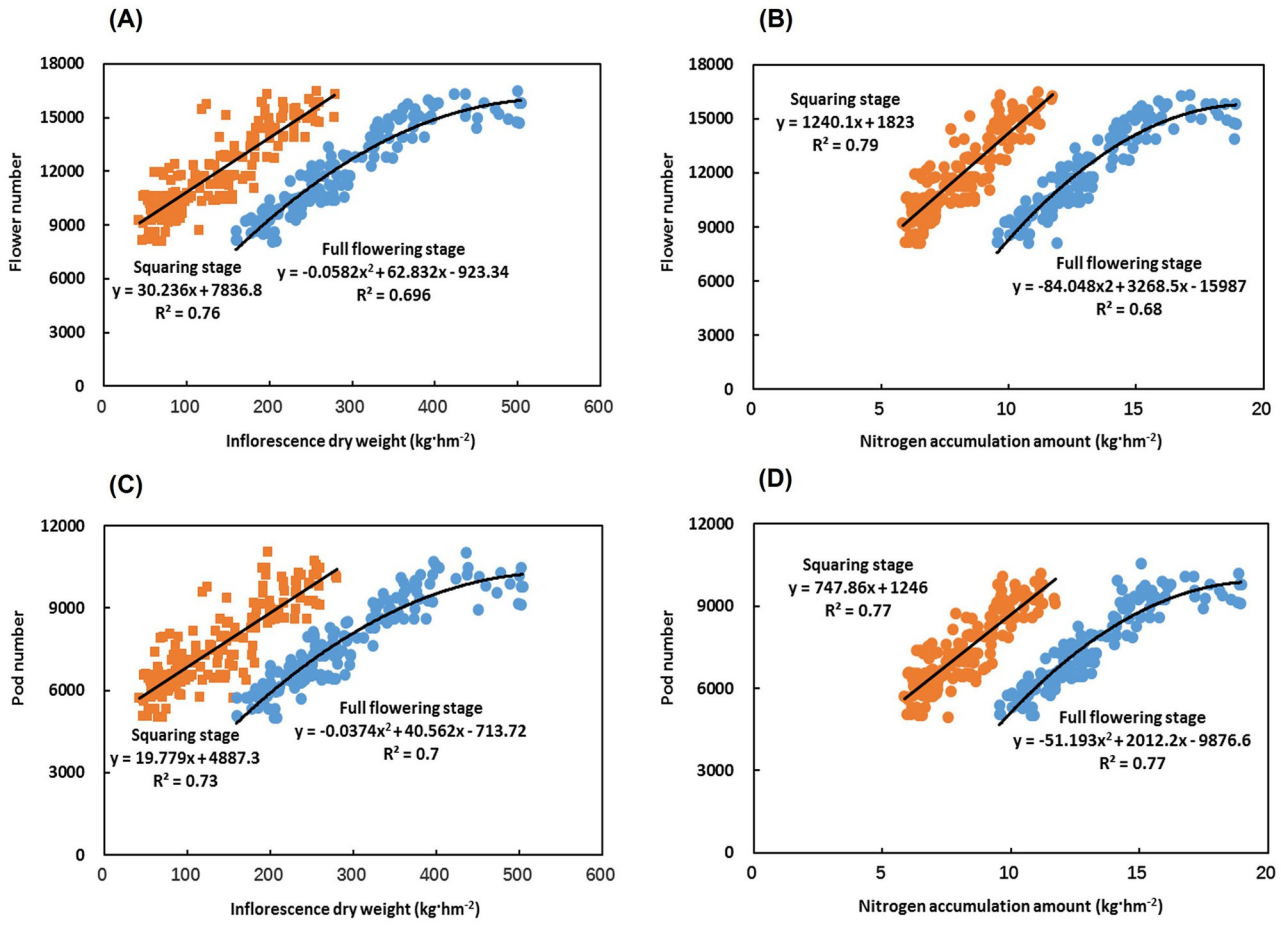
Relationship between the dry matter and nitrogen accumulation in the inflorescence organs of Chinese milk vetch and the number of flowers and pods. (A) Relationship between flower number and inflorescence dry weight of Chinese milk vetch (n = 180). (B) Relationship between flower number and inflorescence nitrogen accumulation in Chinese milk vetch (n = 180). (C) Relationship between pod number and inflorescence dry weight of Chinese milk vetch (n = 180). (D) Relationship between pod number and inflorescence nitrogen accumulation in Chinese milk vetch (n = 180). Orange symbols represent the squaring stage, and blue symbols represent the full flowering stage. All the experiments are shown.

The inflorescence dry weight and nitrogen accumulation at the squaring stage showed a linear correlation with the flower number, and the flower number increased with the increasing dry weight and nitrogen accumulation (Fig 3A and 3B). The coefficients of determination for the equations were 0.76 and 0.79, respectively (*Y = 30*.*236X + 7836*.*8*, *R*^*2*^ *= 0*.*76*, *P<0*.*001*, *n = 180; Y = 1240*.*1X + 1823*, *R*^*2*^ *= 0*.*79*, *P<0*.*001*, *n = 180*) (Fig 3A and 3B). The inflorescence dry weight and nitrogen accumulation at the full flowering stage showed a quadratic relationship with the flower number (Fig 3A and 3B). The flower number first increased and then stabilized with the increasing inflorescence dry weight and nitrogen accumulation (Fig 3A and 3B). The coefficients of determination for the regression equations were 0.696 and 0.68, respectively (*Y = -0*.*0582X*^*2*^ *+ 62*.*832X -923*.*34*, *R*^*2*^ *= 0*.*696*, *P<0*.*001*, *n = 180; Y = -84*.*048X*^*2*^ *+ 3268*.*5X -15987*, *R*^*2*^ *= 0*.*68*, *P<0*.*001*, *n = 180*) (Fig 3A and 3B). The dry weight and nitrogen accumulation of inflorescences at the squaring stage showed linear correlations with the number of pods, and the number of pods increased with the increasing dry weight and nitrogen accumulation, with coefficients of determination of 0.73 and 0.77, respectively (*Y = 19*.*779X + 4887*.*3*, *R*^*2*^ *= 0*.*73*, *P<0*.*001*, *n = 180; Y = 747*.*86X + 1246*, *R*^*2*^ *= 0*.*77*, *P<0*.*001*, *n = 180*) (Fig 3C and 3D). The relationship between the inflorescence dry weight and nitrogen accumulation at the full flowering stage and the number of pods showed a quadratic curve (Fig 3C and 3D). The number of pods first increased and then stabilized with the increasing inflorescence dry weight and nitrogen accumulation (*Y = -0*.*0374X*^*2*^ *+ 40*.*562X -713*.*72*, *R*^*2*^ *= 0*.*7*, *P<0*.*001*, *n = 180; Y = -51*.*193X*^*2*^ *+ 2012*.*2X -9876*.*6*, *R*^*2*^ *= 0*.*77*, *P<0*.*001*, *n = 180*) (Fig 3C and 3D).

### Relationships of seed number with dry matter and nitrogen accumulation in inflorescence organs, nitrogen accumulation in non-inflorescence organs, and the proportion of nitrogen distribution in the inflorescence in Chinese milk vetch

**Fig 4:**
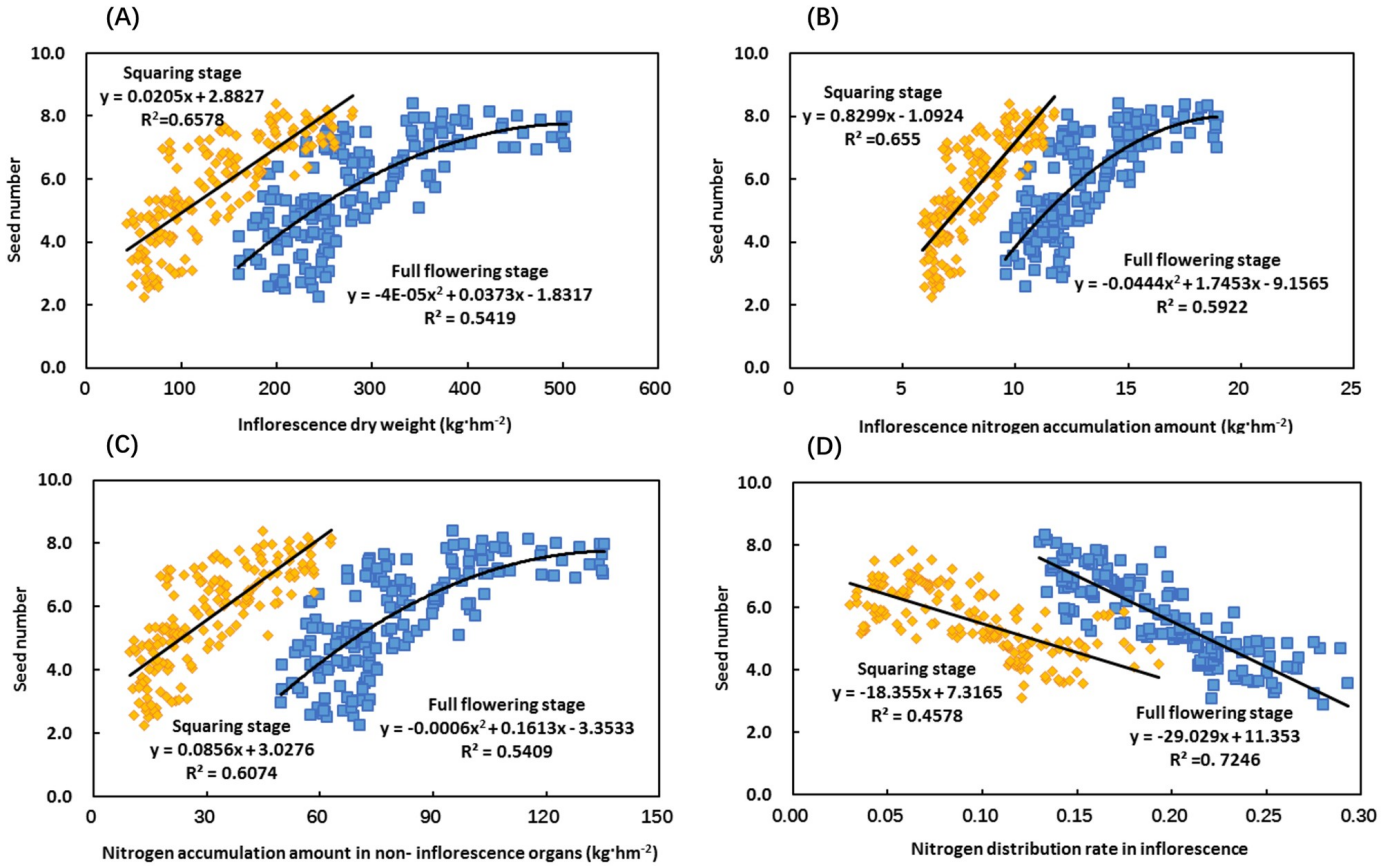
Relationships of seed number with dry matter, nitrogen accumulation and the proportion of nitrogen distribution in inflorescence organs, nitrogen accumulation in non-inflorescence organs in Chinese milk vetch. Relationship between seed number and dry weight, nitrogen accumulation amount and nitrogen distribution rate in inflorescence organs of Chinese milk vetch (n = 180, A, B and D, respectively), and relationship between seed number and nitrogen accumulation amount in non-inflorescence organs of Chinese milk vetch (n = 180, C). Yellow symbols represent the squaring stage, and blue symbols represent the full flowering stage. All the experiments are shown.

The dry matter and nitrogen accumulation in inflorescence organs and nitrogen accumulation in non-inflorescence organs during the squaring stage showed linear correlations with the seed number (Fig 4A–4C). The seed number increased with the increasing dry matter and nitrogen accumulation in inflorescence organs and nitrogen accumulation in non-inflorescence organs. The coefficients of determination for the equation were 0.6578, 0.655, and 0.6074, respectively (*Y = 0*.*0205X +2*.*8827*, *R*^*2*^ *= 0*.*6578*, *P<0*.*001*, *n = 180; Y = 0*.*8299X -1*.*0924*, *R*^*2*^ *= 0*.*655*, *P<0*.*001*, *n = 180*; and *Y = 0*.*0856X +3*.*0276*, *R*^*2*^ *= 0*.*6074*, *P<0*.*001*, *n = 180*) (Fig 4A–4C). The dry matter and nitrogen accumulation in inflorescence organs and the nitrogen accumulation in non-inflorescence organs during the full flowering stage showed quadratic relationships with the seed number (Fig 4A–4C). The seed number first increased and then stabilized with the increasing dry matter and nitrogen accumulation in the inflorescence organs and nitrogen accumulation in the non-inflorescence organs (Fig 4A–4C). The coefficients of determination in the equation were 0.5419, 0.5922, and 0.5409, respectively (*Y = -4E-05X*^*2*^ *+ 0*.*0373X -1*.*8317*, *R*^*2*^ *= 0*.*5419*, *P<0*.*001*, *n = 180; Y = -0*.*0444X*^*2*^ *+ 1*.*7453X -9*.*1565*, *R*^*2*^ *= 0*.*5922*, *P<0*.*001*, *n = 180*; and *Y = -0*.*0006X*^*2*^ *+ 0*.*1613X -3*.*3533*, *R*^*2*^ *= 0*.*5409*, *P<0*.*001*, *n = 180*) (Fig 4A–4C). However, the nitrogen distribution rate in the inflorescences at both the squaring stage and the full flowering stage was negatively correlated with the seed number (Fig 4D). The seed number decreased with the increase in the nitrogen distribution rate in inflorescences (Fig 4D).

### Relationship between dry matter translocation and seed yield of Chinese milk vetch

**Fig 5:**
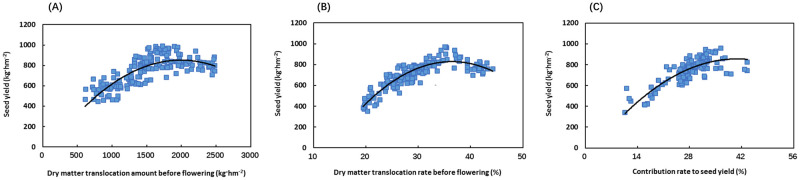
Relationship between dry matter translocation and seed yield of Chinese milk vetch. (A) Relationship between seed yield and dry matter translocation amount of vegetative organs before flowering (*Y = -0*.*0002X*^*2*^ *+ 0*.*9483X – 93*.*309*, *R*^*2*^ *= 0*.*6382*, *P<0*.*001*, *n = 180*). (B) Relationship between seed yield and dry matter translocation rate of vegetative organs before flowering (*Y = -1*.*4937X*^*2*^ *+ 108*.*78X – 1152*.*2*, *R*^*2*^ *= 0*.*7143*, *P<0*.*001*, *n = 180*). (C) Relationship between seed yield and dry matter contribution rate of vegetative organs to seed yield before flowering (*Y = -0*.*5781X*^*2*^ *+ 47*.*142X – 105*.*53*, *R*^*2*^ *= 0*.*6833*, *P<0*.*001*, *n = 180*). All the experiments are shown.

The seed yield of Chinese milk vetch showed a quadratic relationship with the translocation amount and translocation rate of dry matter in the vegetative organs before flowering and their contribution rate to the seed yield (Fig 5A–5C). The seed yield increased first and then decreased with the increasing translocation amount and the translocation rate of dry matter in the vegetative organs before flowering and with their contribution rate to the seed yield (Fig 5A–5C). The coefficients of determination in the regression equations were 0.6382, 0.7143, and 0.6833, respectively (*Y = -0*.*0002X*^*2*^ *+ 0*.*9483X – 93*.*309*, *R*^*2*^ *= 0*.*6382*, *P<0*.*001*, *n = 180; Y = -1*.*4937X*^*2*^ *+ 108*.*78X – 1152*.*2*, *R*^*2*^ *= 0*.*7143*, *P<0*.*001*, *n = 180*; and *Y = -0*.*5781X*^*2*^ *+ 47*.*142X – 105*.*53*, *R*^*2*^ *= 0*.*6833*, *P<0*.*001*, *n = 180*) (Fig 5A–5C). The prediction by the regression equation showed that when the translocation amount of dry matter in the vegetative organs before flowering, the translocation rate of dry matter and the contribution rate to the seed yield reached 1500–1800 kg hm^-2^, 30–35%, and 28–38%, respectively, the seed yield of Chinese milk vetch would reach 800–1000 kg hm^-2^. If the three index values were lower or higher than the above ranges, the seed yield would be lower than 800 kg hm^-2^ (Fig 5A–5C).

### Relationship between nitrogen translocation and seed yield of Chinese milk vetch

**Fig 6:**
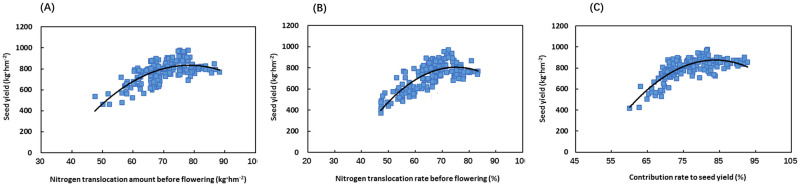
Relationship between nitrogen translocation and seed yield of Chinese milk vetch. (A) Relationship between seed yield and nitrogen translocation amount of vegetative organs before flowering (*Y = -0*.*4571X*^*2*^ *+ 71*.*665X – 1975*.*6*, *R*^*2*^ *= 0*.*494*, *P<0*.*001*, *n = 180*). (B) Relationship between seed yield and nitrogen translocation rate of vegetative organs before flowering (*Y = -0*.*5222X*^*2*^ *+ 78*.*442X − 2140*, *R*^*2*^ *= 0*.*6498*, *P<0*.*001*, *n = 180*). (C) Relationship between seed yield and nitrogen contribution rate of vegetative organs to seed yield before flowering (*Y = -0*.*7965X*^*2*^ *+ 133*.*45X – 4714*.*8*, *R*^*2*^ *= 0*.*6355*, *P<0*.*001*, *n = 180*). All the experiments are shown.

The seed yield of Chinese milk vetch showed a quadratic relationship with the translocation amount and translocation rate of nitrogen in the vegetative organs before flowering and the contribution rate to the seed yield (Fig 6A–6C). The coefficients of determination in the regression equation were 0.494, 0.6498, and 0.6355, respectively (*Y = -0*.*4571X*^*2*^ *+ 71*.*665X – 1975*.*6*, *R*^*2*^ *= 0*.*494*, *P<0*.*001*, *n = 180; Y = -0*.*5222X*^*2*^ *+ 78*.*442X – 2140*, *R*^*2*^ *= 0*.*6498*, *P<0*.*001*, *n = 180*; and *Y = -0*.*7965X*^*2*^ *+ 133*.*45X – 4714*.*8*, *R*^*2*^ *= 0*.*6355*, *P<0*.*001*, *n = 180*) (Fig 6A–6C). The regression equation predicted that when the nitrogen translocation amount of the vegetative organs before flowering, the nitrogen translocation rate, and the contribution rate to the seed yield reached 68–78 kg hm^-2^, 65–75%, and 75–85%, respectively, the seed yield of Chinese milk vetch at the maturity stage would be as high as 800–1000 kg hm^-2^. If the three index values were lower or higher than the above ranges, the seed yield would be lower than 800 kg hm^-2^ (Fig 6A–6C).

## Discussion

### Flower/pod development and seed setting pattern of Chinese milk vetch

There have been many reports on the differentiation and development of floral buds in Fabaceae plants. The morphological differentiation of flower buds is divided into five stages: the undifferentiated stage, the floral primordium differentiation stage, the sepal primordium differentiation stage, the petal primordium differentiation stage, and the stamen primordium and pistil primordium differentiation stage [31, 32]. Bernier *et al*. [33] divided the process into seven stages: the undifferentiated stage, inflorescence primordium differentiation stage, floral primordium differentiation stage, sepal primordium differentiation stage, petal primordium differentiation stage, stamen and pistil primordium differentiation stage, and stamen and pistil structural differentiation stage. Because the time interval between the petal primordium and stamen and the pistil primordium differentiation of Chinese milk vetch is very short, Bagnall [34] grouped them into one stage and ultimately divided the morphological differentiation of the flower buds into six stages, i.e., the flower bud differentiation preparation, inflorescence primordium differentiation, floral primordium differentiation, sepal primordium differentiation, petal primordium differentiation, stamen and pistil primordium differentiation stages, and the pollen and ovule formation stage. In this study, according to the morphological changes in flower/pod development, we divided the flower/pod development process into three stages: flower bud differentiation, flower/pod shedding, and seed setting (Fig 1).

The flowering period of Chinese milk vetch lasts up to 38–43 days. The inflorescences flower in sequence from bottom to top, and pod setting occurs in this same order (Fig 1). The shedding of the flower/pods also occurs in this order. In terms of the shedding ratio, the lower inflorescence generally shows a lower rate of shedding of flower/pods and a higher rate of upper inflorescence [25]. In the current seed production process of Chinese milk vetch, the flower shedding rate is approximately 85%, and the pod shedding rate is approximately 75%, which mostly occurs during the full flowering stage to the end of the flowering stage. The pod setting rate of Chinese milk vetch during the maturity stage is approximately only 50%, and the seed setting rate of pods is less than 90% [35].

### Relationship between the dry matter and nitrogen accumulation of Chinese milk vetch and the development of flowers and pods into seeds

The final manifestation of a series of physiological processes, such as flower/pod development and seed setting, is the key to achieving a high seed number. During flower/pod development, the quality of flower/pod development directly determines the seed number and seed yield [16]. In the current seed production process of Chinese milk vetch, there is a common problem of more flowering but less pod setting and serious flower and pod falling [35]. Zheng *et al*. [27] noted that with the continuous emergence of plant reproductive organs and the continuous, rapid growth of vegetative organs, a shortage of nutrients or an imbalance in the nutrient distribution in the plant body is the main physiological reason for many flowers and pod shedding. Zhong *et al*. [25] indicated that the low pod setting of Chinese milk vetch is caused not only by a tendency toward pod shedding during the maturity stage but also by a large amount of shedding during the flowering and pod setting periods, especially flower shedding. Particular attention should be paid to the preservation of pods and, especially, flowers.

In this study, we analysed the relationship between the dry matter and nitrogen accumulation in the inflorescence of Chinese milk vetch and the number of flowers and pods. The inflorescence dry weight and nitrogen accumulation at the full flowering stage both had quadratic relationships with the numbers of flowers and pods (Fig 3A–3D). This number first increased and then stabilized with the increase in the dry weight of the inflorescence and the amount of nitrogen accumulation (Fig 3A–3D). This result indicates that the dry matter accumulation in the inflorescence organs during this period was high, and the nitrogen accumulation was sufficient, which are conducive to the formation of flowers and pods. However, the accumulation of dry matter and nitrogen should not be too high, as they will lead to excessive growth of the vegetative organs, which is not conducive to nutrient absorption by the inflorescence organs and thus prevent the formation of flowers and pods. The dry weight and nitrogen accumulation of inflorescence organs and the nitrogen accumulation of non-inflorescence organs at the squaring stage showed a linear correlation with the seed number (Fig 4A–4C). However, there was a negative correlation between the nitrogen distribution ratio in the inflorescences and the seed number (Fig 4D). In summary, the accumulation of dry matter and nitrogen in various organs of Chinese milk vetch can directly affect the development of flowers and pods and their morphogenesis: the vigorous growth of inflorescence organs, high dry matter accumulation, and sufficient nitrogen accumulation are conducive to flower, pod, and seed formation. The non-inflorescence organs had high nitrogen accumulation, while the inflorescence organs had a low nitrogen distribution ratio, which was conducive to the increase in the number of seeds.

### Relationship between dry matter and nitrogen accumulation and the translocation in Chinese milk vetch and seed yield

Crop yield is closely related to the accumulation and translocation of dry matter and nitrogen. Analysing the dynamics of the accumulation and translocation of dry matter and nitrogen during crop growth has important practical value in revealing the formation of the crop yield and the construction of high-efficiency populations [21–24]. Research [36, 37] has indicated that the yield of maize is determined by the characteristics of dry matter accumulation and translocation of plants. Improving the dry matter production capacity and the translocation ability of assimilates to the seed and coordinating the source-sink relationship are effective ways to improve yield. We found that the seed yield of Chinese milk vetch showed a quadratic relationship with the dry matter at the full flowering stage, and the seed yield first increased and then decreased with the increase in dry matter (Fig 2C), indicating that the dry matter accumulation of Chinese milk vetch during the full flowering stage should not be too large, because otherwise it will lead to an increase in ineffective branches, which is not conducive to the establishment of a balanced ecological structure in the population. The appropriate amount of dry matter accumulation during this period is the basis for high yield formation.

A correlation analysis of Chinese milk vetch seed yield and seed-setting factors showed that there was a positive correlation between the seed yield and the number of inflorescences and pods, pod setting rate, and seed number (Table 1). By analysing the relationship between dry matter accumulation and the seed number during the growth stage of Chinese milk vetch, we found that the dry matter at the early flowering stage and the dry matter accumulation at the elongation to early flowering stages were all linearly correlated with the seed number (Fig 2A and 2B). The seed number increased with the increase in dry matter accumulation at the early flowering stage and the elongation to early flowering stages (Fig 2A and 2B), indicating that under increased yield conditions, a rapid and high accumulation of dry matter during the spring is the material basis for good seed development.

The rational distribution and utilization of crop dry matter and nitrogen between sources and sinks is critical to maximizing the yield potential [38, 39]. In this study, by modelling the relationship between the dry matter and nitrogen translocation of Chinese milk vetch and the seed yield, it was found that when the translocation of stored assimilates in the vegetative organs before flowering, the assimilate translocation rate, and the contribution rate to the seed yield reached 1500–1800 kg hm^-2^, 30–35%, and 28–38%, respectively, the seed yield of Chinese milk vetch reached 800–1000 kg hm^-2^ at the maturity stage (Fig 5A–5C); when the translocation of nitrogen in the vegetative organs before flowering, the nutrient translocation rate, and the contribution rate to the seed yield reached 68–78 kg hm^-2^, 65–75%, and 75–85%, the seed yield of Chinese milk vetch at the maturity stage reached 800–1000 kg hm^-2^ (Fig 6A–6C). If the translocation indices of dry matter and nitrogen are lower or higher than the above ranges, the seed yield will be lower than 800 kg hm^-2^. By quantifying the relationships that prevail during the seed production of Chinese milk vetch, the source-sink relationship can be improved by optimizing cultivation measures during the critical period of flower and pod development, thereby coordinating the distribution of dry matter and nitrogen between the source and sink and achieving high yield, stable yield, and high-quality Chinese milk vetch products.

## Conclusions

This study analysed the relationship between the seed yield of Chinese milk vetch and seed-setting factors and found that the seed yield of Chinese milk vetch was positively correlated with the number of inflorescences and pods, the seed setting rate of pods, and the seed number but not with the number of plants, number of branches, or thousand-seed weight (Table 1). Through the analysis and quantification of the relationship between the dry matter, nitrogen accumulation and translocation of Chinese milk vetch and the seed setting and seed yield, it was found that strengthening the population quality, stabilizing the dry matter quality at the full flowering stage, enhancing the dry matter growth rate in spring, reducing the nitrogen distribution ratio in the inflorescences, stabilizing the translocation amounts and translocation rates of dry matter and nitrogen in the vegetative organs before flowering, and enhancing the dry matter and nitrogen translocation capacity of the vegetative organs to the seed can promote seed setting and increase the seed yield of Chinese milk vetch.

## Supporting information

S1 Dataset Relevant data underlying the findings described in this manuscript. (XLS)
